# Characteristics and Outcomes of Heart Failure Patients from a Middle-Income Country: The RECOLFACA Registry

**DOI:** 10.5334/gh.1145

**Published:** 2022-08-18

**Authors:** Juan Esteban Gomez-Mesa, Clara Saldarriaga, Luis Eduardo Echeverría, Alex Rivera-Toquica, Paula Luna, Sebastián Campbell, Lisbeth Natalia Morales, Juan David López Ponce de León, Andrés Felipe Buitrago, Erika Martínez, Jorge Alberto Sandoval, Alexis Llamas, Gustavo Adolfo Moreno, Julián Vanegas, Fernán Mendoza Beltrán

**Affiliations:** 1 Fundación Valle de Lili, Cali, Colombia; 2 Universidad Icesi, Cali, Colombia; 3 Asociación Sociedad Colombiana de Cardiología y Cirugía Cardiovascular (SCC), CO; 4 Sociedad Interamericana de Cardiología (SIAC), Colombia; 5 Clínica Cardio Vid, Medellín, Colombia; 6 Universidad de Antioquia, Medellín, Colombia; 7 Fundación Cardiovascular de Colombia, Floridablanca, Colombia; 8 Clínica los Rosales, Pereira, Colombia; 9 Universidad Tecnológica de Pereira, Pereira, Colombia; 10 Clínica Medilaser, Florencia, Colombia; 11 Clínica Medilaser, Tunja, Colombia; 12 Fundación Santa Fe, Bogotá, Colombia; 13 IPS Salud Social, Barranquilla, Colombia; 14 Cardiología Siglo XXI, Ibagué, Colombia; 15 Clínica Las Américas, Medellín, Colombia; 16 Hospicardio, Montería, Colombia; 17 Instituto Cardiovascular Colombiano, Manizales, Colombia; 18 Fundación Clínica Shaio, CO

**Keywords:** Heart Failure, registries, Latin America

## Abstract

**Background:**

There is a lack of epidemiological data around heart failure (HF) in Latin America; the potential impact description of this disease in middle-income countries is relevant.

**Objective:**

This study aimed to describe the characteristics and healthcare resource utilization patterns of HF patients at baseline and six-month follow-up.

**Methods:**

This retrospective observational study used data from the RECOLFACA (*Registro Colombiano de Falla Cardíaca*) registry, which includes data obtained from the examination of clinical records from 2,528 patients in 60 Colombian healthcare institutions. Baseline and six-month follow-up data were evaluated from patients with previous hospital admissions due to HF during the 12 months prior to enrollment.

**Results:**

This study analyzed 2,045 patients (42.8% female) with a mean age of 67.71 ± 13.64 years. The most common etiologies were ischemic (44.4%) and hypertensive heart disease (38.5%). At baseline, 53.4% of patients were classified with NYHA class II, and 73.6% had a reduced left ventricle ejection fraction (LVEF). A year prior to entering the registry, patients were hospitalized an average of 1.4 ± 1.1 times due to HF. Prescription of evidence-based treatment at baseline included sacubitril/valsartan (10%), ACEI (33%), ARB (41%), beta-blocker (79%), diuretics (68%), and MRA (56%). The average quality of life score measured using the EQ-5D-3L questionnaire was 78.7 ± 20.8 at baseline and 82.3 ± 20.1 at the six-month follow-up. The mortality rate was 6.7%.

**Conclusions:**

The use of information from the RECOLFACA registry allowed characterization as well as analyses of healthcare resource utilization of patients with heart failure in Colombia. The results of this study show that multiple evidence-based treatments for HF are being widely used in Colombia, but there seems to be room for improvement regarding some interventions for the treatment of patients with HF.

## Introduction

Heart failure (HF) is a global public health problem. In Latin America (LA), most of the epidemiology of HF relies on data from Europe and North America. However, its prevalence is estimated to be around 1%, and it is expected to increase in the following years [1]. Still, the magnitude of the burden of disease of HF cannot be assessed with precision because reliable population-based studies are lacking.

Early post-discharge mortality and readmission rates remain high, and many patients have poor long-term survival, even with contemporary management and available pharmacological treatments [2,3]. A worse prognosis can occur in Latin American countries, as there are differences in HF severity, etiology, and management, potentially leading to substantial differences in health outcomes. Previous research has reported that South American patients had higher overall mortality than other world regions [4].

In Colombia, HF is also a public health concern of particular importance. The country faces many of the risk factors seen in developed countries and a high prevalence of Chagas disease, which contributes significantly to the national burden of cardiovascular disease [5]. Additionally, there is disparity in the distribution of healthcare services in the different regions of Colombia [6]. Even though healthcare coverage in Colombia for 2019 was 95% ensured either by the contributive regime (includes mandatory payments from employers) or by subsidized regime (set up initially for people outside the formal sector and with very low income) [7], in regions such as Orinoco or Amazonas, health coverage falls under 70% [8,9]. Despite the fact that HF medication is guaranteed in the health benefit program [10], information regarding the health resource utilization associated with this condition in Colombia is scarce, and there is little evidence related to the epidemiology of HF in Colombia [11,12]. That is why a national HF registry can provide valuable epidemiological data. Moreover, it can contribute to a better understanding of this syndrome and its local management.

This study aimed to fill the existing evidence gap by describing the demographic, clinical characteristics, QoL, and healthcare resource utilization patterns of chronic HF patients from the RECOLFACA registry, including 60 hospitals in 29 different Colombian cities. To our knowledge, RECOLFACA is the largest registry in Colombia, which provides valued epidemiological evidence and uncovers the current clinical practice for HF management in Colombia.

## Methods

### Design

We conducted a retrospective observational study using data from 2016 to 2020 from the RECOLFACA registry. The division of Heart Failure, Pulmonary Hypertension and Heart Transplant of the Colombian Cardiology Society (SCC, *Sociedad Colombiana de Cardiología y Cirugía Cardiovascular*) created this registry. Researchers collected and consolidated the data in an online platform (INFAMED) available in each institution. The study received ethical approval from the Fundación Valle del Lili IRB (*Comité de Ética en Investigación Biomédica IRB*), and patients gave their written informed consent to participate at enrollment.

### Data source, data quality control, and statistical considerations

The RECOLFACA database is a registry of 2,528 patients with HF and more than 90 variables measured. It was created in 2016 and consisted of two phases of data collection: In the first phase (phase I, 2016–2018), 20 institutions in 11 Colombian cities participated in the recruitment process, whereas in the second phase (phase II: 2018–2020), 40 additional institutions from 18 additional cities joined the registry to assure representation of the five Colombian regions. The registry collected data from patients at two moments: At baseline, that is, when they were enrolled, and six months later, as a follow-up. The registry includes sociodemographic information, medical history, information regarding in-patient visits, medication use, etiology of HF (determined by primary physician), assessment of HF, treatment for HF at baseline and follow-up, clinical outcomes at follow-up (decompensation, EKG, %LVEF, biochemical markers), and quality of life (QoL) assessment. For this last variable, the EQ-5D-3L questionnaire, which measures patients’ mobility, ability to self-care, ability to carry out usual activities, grade of pain or discomfort, and presence of anxiety or depression [13], was used due to it being preferred by physicians in Colombia and its availability in the databases.

This study included adult patients (≥18 years old) with a history of hospital admission due to heart failure during the 12 months prior to study baseline who were currently attending cardiology or heart failure–related medical consultations at a healthcare institution participating in the RECOLFACA registry. Patients who had a history of cardiac transplant or were on a cardiac transplant waiting list were excluded. Likewise, patients who had a history of ventricular assistance device (VAD) implantation, who were on a waiting list for VAD implantation, or who had a neurological or social disability that would limit the follow-up were not eligible for the study.

This database was set and cleaned using R Statistical Software [14], ensuring adherence to all local and regional laws on data protection and privacy. We conducted the statistical analyses after checking the data set for quality issues and missing variables. We included only patients with available data at baseline and the six-month follow-up. Furthermore, patients with missing data for relevant variables, such as gender and age, and for clinical characteristics at baseline, such as left ventricular ejection fraction (LVEF), stage of heart failure, and NYHA functional classification, were excluded from the analyses. No data imputation for variables with null values was conducted.

We described all variables according to their type. We employed frequencies and proportions for categorical variables, and we used central tendency statistics (mean, median) and dispersion measures (variance, standard deviation) for continuous variables. Analysis of change from baseline was performed for clinical outcomes. All statistical analyses were conducted using R Statistical Software [14]. The study was conducted in accordance with the revised guidelines of the World Medical Association Declaration of Helsinki and local laws and regulations.

## Results

### Demographic characteristics

**Figure 1:**
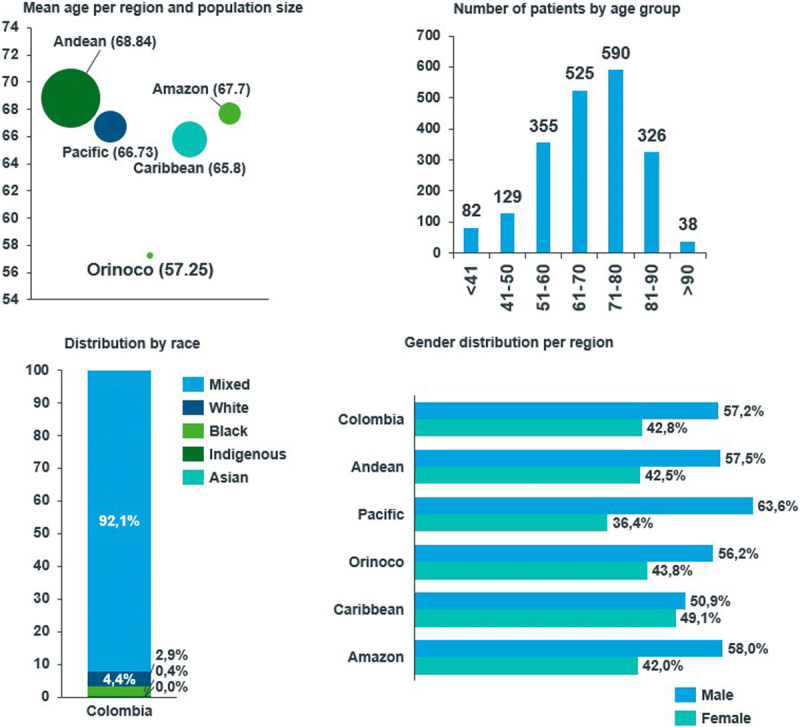
Demographic characteristics of patients from the RECOLFACA registry.

Our analysis included 2,045 patients from this registry from Colombia who met the eligibility criteria. Table 1 and Figure 1 show an overview of the demographic characteristics of the patients. The mean age of the population was 67.71 ± 13.64 years, with patients from the Orinoco region being younger than patients from the rest of the country (57.25 ± 11.53). The patient population mainly included males (57.2%). Most patients in the registry had a mixed race or ethnic background. In the Pacific region, a higher proportion of the population was black (13.4%), while 6% of the Amazon and Pacific regions were indigenous.

**Table 1:** Demographic characteristics of patients from the RECOLFACA registry at baseline.


AGE (YEARS)	COLOMBIA	ANDEAN	PACIFIC	ORINOCO	CARIBBEAN	AMAZON

**Mean ± SD**	67.71 ± 13.64	68.84 ± 12.92	66.73 ± 14.53	57.25 ± 11.53	65.80 ± 14.01	67.70 ± 14.67

**Gender *N* (%)**						

**Female**	875 (42.8)	481 (42.5)	122 (36.4)	7 (43.8)	199 (49.1)	66 (42.0)

**Male**	1,170 (57.2)	651 (57.5)	213 (63.6)	9 (56.2)	206 (50.9)	91 (58.0)

**Race *N* (%)**						

**Mixed**	1,884 (92.1)	1,075 (95.0)	265 (79.1)	16 (100.0)	381 (94.1)	147 (93.6)

**White**	91 (4.4)	51 (4.5)	19 (5.7)	0 (0.0)	12 (3.0)	9 (5.7)

**Black**	60 (2.9)	3 (0.3)	45 (13.4)	0 (0.0)	12 (3.0)	0 (0.0)

**Indigenous**	9 (0.4)	2 (0.2)	6 (1.8)	0 (0.0)	0 (0.0)	1 (0.6)

**Asian**	1 (0.0)	1 (0.1)	0 (0.0)	0 (0.0)	0 (0.0)	0 (0.0)

**Schooling *N* (%)**						

**Basic primary school**	782 (38.2)	448 (39.6)	141 (42.1)	6 (37.5)	128 (31.6)	59 (37.6)

**High school**	558 (27.3)	308 (27.2)	105 (31.3)	6 (37.5)	110 (27.2)	29 (18.5)

**None**	385 (18.8)	173 (15.3)	31 (9.3)	2 (12.5)	118 (29.1)	61 (38.9)

**Technical/technological education**	150 (7.3)	84 (7.4)	37 (11.0)	1 (6.2)	26 (6.4)	2 (1.3)

**University/professional education**	147 (7.2)	106 (9.4)	15 (4.5)	1 (6.2)	19 (4.7)	6 (3.8)

**Postgraduate education**	23 (1.1)	13 (1.1)	6 (1.8)	0 (0.0)	4 (1.0)	0 (0.0)

**Type of health insurance *N* (%)**						

**Contributory**	1,182 (57.8)	713 (63.0)	260 (77.6)	8 (50.0)	130 (32.1)	71 (45.2)

**Subsidized**	733 (35.8)	316 (27.9)	55 (16.4)	7 (43.8)	273 (67.4)	82 (52.2)

**Additional health insurance policy**	130 (6.4)	103 (9.1)	20 (6.0)	1 (6.2)	2 (0.5)	4 (2.5)

**Zone *N* (%)**						

**Rural**	560 (27.4)	308 (27.2)	64 (19.1)	6 (37.5)	135 (33.3)	47 (29.9)

**Urban**	1,485 (72.6)	824 (72.8)	271 (80.9)	10 (62.5)	270 (66.7)	110 (70.1)


The results show that most of the population had low education levels, with 38.2% having only reached basic primary school and 18.8% having no formal education. The proportion of patients from the registry living in urban areas was high (72.6%), and more than half of the patients (57.8%) were enrolled in the national contributive insurance scheme.

### Heart failure etiology

**Figure 2:**
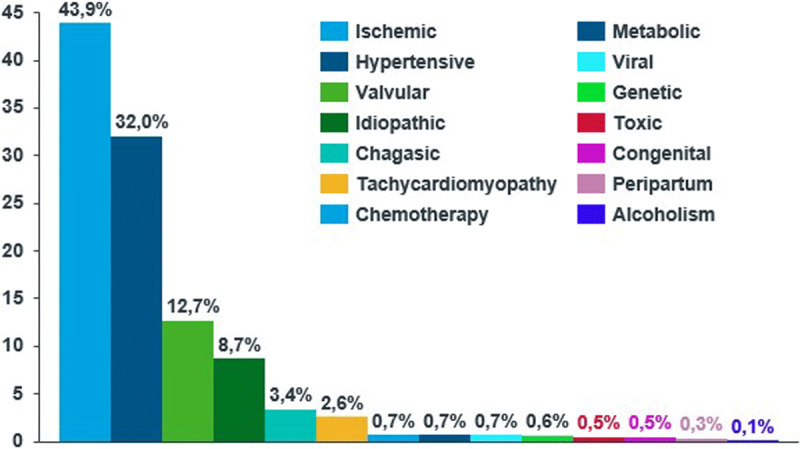
Etiology of heart failure.

The most common etiology of HF was ischemic heart disease (43.9%), followed by hypertensive heart disease (32.0%) and valvular disease (12.7%). Chagas disease was reported in 3.4% of the patients. A higher proportion of patients from the Andean region had been diagnosed with this disease (53 patients). Figure 2 shows the different HF etiologies of patients from the RECOLFACA registry.

### Comorbidities

The most frequent associated comorbidities found in patients from the registry were hypertension (72.2%), followed by diabetes (27.1%), and dyslipidemia (26.9%).

### Hospital admission history

In agreement with the inclusion criteria, all patients required at least one prior hospitalization to be included in the registry. The most common cause of hospitalization was, exclusively, acute heart failure (80.3%) (Table 2).

**Table 2:** Hospital admission history.


HOSPITAL ADMISSION HISTORY	BASELINE (LAST YEAR) *N* = 2,045						6-MONTH FOLLOW-UP (LAST 6 MONTHS) *N* = 1,907*					
	
	COLOMBIA	ANDEAN	PACIFIC	ORINOCO	CARIBBEAN	AMAZON	COLOMBIA	ANDEAN	PACIFIC	ORINOCO	CARIBBEAN	AMAZON

**Due to heart failure *N* (%)**	2,045 (100.0)	1,132 (100.0)	335 (100.0)	16 (100.0)	405 (100.0)	157 (100.0)	462 (24.2)	231 (22.1)	82 (25.9)	3 (18.8)	109 (28.3)	37 (25.9)

**Only due to HF *N* (%)**	1,643 (80.3)	916 (80.9)	284 (84.8)	13 (81.2)	338 (83.5)	92 (58.6)	381 (20.0)	186 (17.8)	69 (21.8)	3 (18.8)	88 (22.9)	35 (24.5)

**Due to HF and other *N* (%)**	402 (19.7)	216 (19.1)	51 (15.2)	3 (18.8)	67 (16.5)	65 (41.4)	81 (4.25)	45 (4.3)	13 (4.1)	2 (1.4)	21 (5.5)	2 (1.4)

**Missing data N (%)**	0 (0.0)	0 (0.0)	0 (0.0)	0 (0.0)	0 (0.0)	0 (0.0)	1,445 (75.8)	815 (77.9)	235 (74.1)	106 (74.1)	276 (71.7)	13 (81.3)

**NUMBER OF HF-RELATED HOSPITALIZATIONS**	**COLOMBIA**	**ANDEAN**	**PACIFIC**	**ORINOCO**	**CARIBBEAN**	**AMAZON**	**COLOMBIA**	**ANDEAN**	**PACIFIC**	**ORINOCO**	**CARIBBEAN**	**AMAZON**

**Mean ± SD**	1.4 ± 1.1	1.3 ± 0.8	1.5 ± 1.3	1.3 ± 1.0	1.6 ± 1.6	1.6 ± 1.3	1.7 ± 2.1	1.8 ± 2.7	1.6 ± 1.4	1.0 ± 0.0	1.6 ± 1.1	1.9 ± 1.3

**Missing data *N* (%)**	0 (0.0)	0 (0.0)	0 (0.0)	0 (0.0)	0 (0.0)	0 (0.0)	1,446 (75.8)	816 (78)	235 (74.1)	13 (81.3)	276 (71.7)	106 (74.1)

**LOS IN HOSPITALIZATION**	**COLOMBIA**	**ANDEAN**	**PACIFIC**	**ORINOCO**	**CARIBBEAN**	**AMAZON**	**COLOMBIA**	**ANDEAN**	**PACIFIC**	**ORINOCO**	**CARIBBEAN**	**AMAZON**

**Mean ± SD**	11.2 ± 12.6	11.7 ± 11.1	11.7 ± 16.3	9.6 ± 7.1	10.3 ± 9.1	9.0 ± 18.3	10.7 ± 9.9	12.3 ± 10.9	9.5 ± 11.8	20.3 ± 12.4	9.5 ± 5.5	8.6 ± 9.4

**Missing data *N* (%)**	859 (42)	496 (43.8)	105 (31.3)	7 (43.8)	179 (44.2)	72 (45.9)	1,644 (86.2)	929 (88.8)	274 (86.4)	13 (81.3)	321 (83.4)	107 (74.8)


HF: heart failure; LOS: length of stay. * Percentages were calculated over the total population that remained alive at the 6-month follow-up.

A year prior to entering the registry, patients were hospitalized an average of 1.4 ± 1.1 times (length of hospital stay of 11.2 ± 12.6 days). At the six-month follow-up, patients had an average of 1.7 ± 2.1 hospitalizations (length of hospital stay of 10.7 ± 9.9 days). Missing data were significant at follow-up for the number of readmissions and the length of hospital stay. Table 2 presents details of hospitalization history at baseline and follow-up.

### Clinical characteristics and outcomes

**Figure 3:**
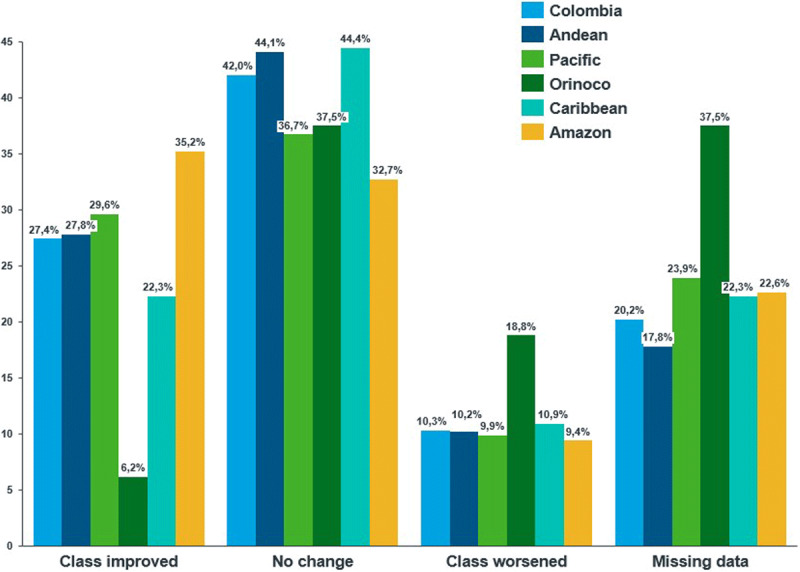
Changes in functional class according to NYHA at baseline.

Based on the New York Heart Association (NYHA) Functional Classification, at baseline, 53.4% of the patients had mild symptoms and slight limitations for ordinary activities (class II), 29.8% of the patients suffered from marked limitations due to symptoms even during less-than-ordinary activity (class III), and 4.8% of the patients were only comfortable at rest (class IV). Twelve percent of the patients had no symptoms and no limitations in ordinary physical activity (class I). As shown in Figure 3, at the six-month follow-up, the functional class of 27.4% of patients improved, while 10.3% worsened NYHA classification.

The average LVEF was 34.2 ± 13.5 at baseline, with 73.6% of the patients having a LVEF ≤40% (heart failure with a reduced ejection fraction, HFrEF). At the six-month follow-up, the average LVEF was 36.7 ± 13.8, and 65.7% of the patients with available ejection fraction at follow-up had HFrEF. Table 3 includes other clinical variables measured at baseline and follow-up, including the American College of Cardiology (ACC) and the American Heart Association (AHA) HF classification and brain natriuretic peptides (BNP/NT pro BNP). Other paraclinical studies are included in Supplementary Table 1.

**Table 3:** Clinical outcomes for patients from the RECOLFACA registry.


	BASELINE (*N* = 2,045)						6-MONTH FOLLOW-UP (*N* = 1,907*)					
	
	COLOMBIA	ANDEAN	PACIFIC	ORINOCO	CARIBBEAN	AMAZON	COLOMBIA	ANDEAN	PACIFIC	ORINOCO	CARIBBEAN	AMAZON

**NYHA classification; *N* (%)**												

**Class I**	245 (12.0)	146 (12.9)	53 (15.8)	3 (18.8)	41 (10.1)	2 (1.3)	386 (20.24)	217 (20.75)	95 (29.97)	4 (25)	43 (11.17)	27 (18.88)

**Class II**	1,092 (53.4)	602 (53.2)	175 (52.2)	12 (75.0)	230 (56.8)	73 (46.5)	941 (49.34)	559 (53.44)	105 (33.12)	3 (18.75)	210 (54.55)	64 (44.76)

**Class III**	609 (29.8)	347 (30.7)	84 (25.1)	1 (6.2)	124 (30.6)	53 (33.8)	262 (13.74)	140 (13.38)	47 (14.83)	3 (18.75)	53 (13.77)	19 (13.29)

**Class IV**	99 (4.8)	37 (3.3)	23 (6.9)	0 (0.0)	10 (2.5)	29 (18.5)	42 (2.2)	14 (1.34)	8 (2.52)	0 (0)	8 (2.08)	12 (8.39)

**Missing data**	0 (0)	0 (0)	0 (0)	0 (0)	0 (0)	0 (0)	276 (14.47)	116 (11.09)	62 (19.56)	6 (37.5)	71 (18.44)	21 (14.69)

**ACA/AHA Failure Stages; n (%)**												

**Stage C**	1,946 (95.2)	1,092 (96.5)	304 (90.7)	16 (100.0)	385 (95.1)	149 (94.9)	1,308 (68.59)	817 (78.11)	207 (65.3)	4 (25)	43 (11.17)	27 (18.88)

**Stage D**	99 (4.8)	40 (3.5)	31 (9.3)	0 (0)	20 (4.9)	8 (5.1)	97 (5.09)	42 (4.02)	26 (8.2)	3 (18.75)	210 (54.55)	64 (44.76)

**Missing data**	0 (0)	0 (0)	0 (0)	0 (0)	0 (0)	0 (0)	226 (11.85)	71 (6.79)	22 (6.94)	3 (18.75)	53 (13.77)	19 (13.29)

**% LEVF**												

**Mean ± SD**	34.2 ± 13.5	34.4 ± 4.2	31.1 ± 12.5	31.9 ± 14.7	33.1 ± 10.9	42.4 ±12.7	36.7 ±13.8	37.5 ± 14.4	32.8 ± 13.0	41.0 ± 22.9	34.9 ± 11.1	43.7 ± 13.8

**Missing data; *N* (%)**	0 (0)	0 (0)	0 (0)	0 (0)	0 (0)	0 (0)	1,144 (59.99)	583 (55.74)	202 (63.72)	13 (81.25)	244 (63.38)	102 (71.33)

**NT-pro BNP (pg/mL)**												

**Mean ± SD**	5,514.0 ± 8,360.7	5,711.9 ± 8,461.4	6,146.6 ± 8,631.1	3,141.7 ± 3,362.7	5,128.4 ± 8,299.6	2,702.5 ± 2,717.4	3,625.4 ± 6,638.4	4,017.2 ± 8,893.5	6,472.3 ± 8,121.5	2,969.5 ± 3,278.9	2,874.9 ± 3,905.3	487.3 ± 504.2

**Missing data; *N* (%)**	1,680 (82.2)	963 (85.1)	283 (84.5)	13 (81.3)	266 (65.7)	155 (98.7)	1,703 (89.3)	972 (92.93)	296 (93.38)	14 (87.5)	281 (72.99)	140 (97.9)

**BNP (pg/mL)**												

**Mean ± SD**	1,616.8 ± 2,232.7	1,593.3 ± 2,312.0	1,386.2 ± 1,222.7	1,097.5 ± 1,450.3	2,179.1 ± 1,745.5	—	1,493.7 ± 2,214.3	1,484.0 ± 2,258.5	8,88.7 ± 1,217.2	23.0 ± nan	3,119.0 ± 2,340.4	560.9 ± nan

**Missing data; *N* (%)**	1,907 (93.3)	1,010 (89.2)	330 (87.5)	14 (87.5)	396 (97.8)	157 (100)	1,832 (96.07)	979 (93.59)	314 (99.05)	15 (93.75)	382 (99.22)	142 (99.3)


* Percentages were calculated over the total population that remained alive at the 6-month follow-up.

### Mortality

As shown in Table 4, the mortality rate was 6.7%, with patients from the Amazon region having the highest rate (8,8%). Most deaths in the registry were cardiovascular deaths (74.6%), with a mean time from recruitment to death of 149.6 ± 128.3 days. By HF etiology, chemotherapy had the highest mortality frequency (25%), followed by toxic (15.8%), Chagas disease (10%), valvular disease (8.4%), and hypertension (7.6%).

**Table 4:** Mortality at six-month follow-up of patients from the RECOLFACA registry.


	COLOMBIA	ANDEAN	PACIFIC	ORINOCO	CARIBBEAN	AMAZON

**Mortality at 6-month follow-up; *N* (%)**	138 (6.7)	86 (7.6)	18 (5.4)	0 (0.0)	20 (5.0)	14 (8.8)

**Cardiovascular deaths; *N* (%)**	103 (74.6)	66 (76.7)	15 (83.3)	0 (0.0)	18 (90.0)	4 (28.6)

**Non-cardiovascular deaths; *N* (%)**	35 (25.4)	20 (23.3)	3 (16.7)	0 (0.0)	2 (10.0)	10 (71.4)

**Mean days of follow-up from start date to death (Mean ± SD)**	149.6 ± 128.3	142.7 ± 129.4	189.3 ± 164.3	0 (0.0)	124.8 ± 76.8	186.3 ± 135.1


### Health-related quality of life

The average QoL score was measured using the EQ-5D-3L questionnaire. The results show a QoL of 78.7 ± 20.8 at baseline and of 82.3 ± 20.1 at the six-month follow-up, with a mean change from baseline of 10.0 ± 60.2. As shown in Table 5, changes from baseline were highly variable between regions, with the Amazon region showing a mean change from baseline of –10.7 ± 38.6 and the Andean region showing a mean change of 18.0 ± 73.3.

**Table 5:** Health-related quality of life score.


	BASELINE MEAN ± SD	6-MONTH FOLLOW-UP MEAN ± SD	MEAN CHANGE FROM BASELINE	MISSING DATA (BASELINE) *N* (%)	MISSING DATA (FOLLOW-UP) *N* (%) *

**Colombia**	78.7 ± 20.8	82.3 ± 20.1	10.0 ± 60.2	0 (0)	279 (14.63)

**Andean**	76.2 ± 21.7	83.6 ± 19.0	18.0 ± 73.3	0 (0)	118 (11.28)

**Pacific**	82.7 ± 19.6	82.2 ± 21.2	2.3 ± 37.8	0 (0)	62 (19.56)

**Orinoco**	84.4 ± 17.5	90.0 ± 15.6	4.8 ± 19.6	0 (0)	6 (37.5)

**Caribbean**	82.6 ± 18.7	84.0 ± 17.8	0.8 ± 26.6	0 (0)	72 (18.7)

**Amazon**	77.0 ± 19.9	68.1 ± 25.9	–10.7 ± 38.6	0 (0)	21 (14.69)


* Percentages were calculated over the total population that remained alive at the 6-month follow-up (*N* = 1,907).

### Treatment patterns

Beta-blockers were the most extensively used therapeutic group at baseline (79% of patients), with carvedilol as the predominantly prescribed medication (63% of patients); see Table 6.

**Table 6:** Medication patterns—RECOLFACA registry.


MEDICATION CLASS	BASELINE			6-MONTH FOLLOW-UP		
	
	TOTAL *N* = 2,045	HFREF *N* = 1,506	LVEF >40% *N* = 539	TOTAL *N* = 1,629*	HFREF *N* = 501***	LVEF >40% *N* = 262***

**Beta-Blocker**							

**Carvedilol; *N* (%)**	1,295 (63.0)	1,019 (67.7)	276 (51.2)	1,065 (65.4)	321 (64.1)	151 (57.6)

**Metoprolol Succinate; *N* (%)**	311 (15.0)	209 (13.9)	102 (18.9)	278 (17.1)	95 (19)	55 (21)

**Nebivolol; *N* (%)**	21 (1.0)	13 (0.9)	8 (1.5)	19 (1.2)	5 (1)	3 (1.2)

**ARNI**						

**Sacubitril/Valsartan; *N* (%)**	203 (10.0)	187 (12.4)	16 (3)	281 (17.2)	121 (24.2)	30 (11.5)

**ACEi**						

**Enalapril; *N* (%)**	677 (33.0)	542 (36)	135 (25.1)	484 (29.7)	165 (32.9)	56 (21.4)

**Captopril; *N* (%)**	6 (0.0)	6 (0.4)	–	3 (0.2)	–	1 (0.49)

**ARB**						

**Losartan; *N* (%)**	745 (36.0)	503 (33.4)	242 (44.9)	554 (34)	138 (27.5)	104 (39.7)

**Valsartan; *N* (%)**	63 (3.0)	32 (2.1)	31 (5.8)	50 (3.1)	13 (2.6)	16 (6.1)

**Candesartan; *N* (%)**	46 (2.0)	30 (2)	16 (3)	34 (2.1)	7 (1.4)	14 (5.3)

**Diuretics**						

**Furosemide; *N* (%)**	1,315 (64.0)	1,033 (68.6)	282 (52.3)	1,018 (62.5) **	341 (68.1)	118 (45)

**Hydrochlorothiazide; *N* (%)**	74 (4.0)	35 (2.3)	39 (7.2)	48 (2.9) **	6 (1.2)	14 (5.3)

**Indapamide; *N* (%)**	5 (0.0)	–	1 (25.0)	4 (0.2) **	–	1 (0.4)

**MRAs**						

**Spironolactone; *N* (%)**	1,091 (53.0)	949 (63)	142 (26.4)	933 (57.3)	336 (67.1)	99 (37.8)

**Eplerenone; *N* (%)**	65 (3.0)	52 (3.5)	13 (2.4)	70 (4.3)	27 (5.4)	15 (5.7)

**Other medication**						

**Antiplatelet medication; *N* (%)**	958 (46.8)	699 (46.4)	259 (48.1)	728 (44.7)	269 (53.7)	133 (64.6)

**Statins; *N* (%)**	1,128 (55.2)	817 (54.3)	311 (57.7)	961 (59)	319 (63.7)	184 (89.3)

**Digoxin; *N* (%)**	204 (10.0)	179 (11.9)	25 (4.6)	174 (10.7)	62 (12.4)	13 (5)

**Ivabradine; *N* (%)**	135 (7.0)	122 (8.1)	13 (2.4)	121 (7.4)	55 (11)	20 (7.6)

**Nitrates; *N* (%)**	83 (4.1)	60 (4.0)	23 (4.3)	51 (3.1)	17 (3.4)	8 (3.9)


HFrEF: heart failure with a reduced ejection fraction (≤40%). * Patients with available data at follow-up; ** *N* = 1,628 at follow-up (Patients with available data for diuretic medication). *** Patients with available ejection fraction at follow-up.

Regarding renin-angiotensin-aldosterone system (RAAS) inhibitors, a third of the patients received ACEi at baseline with enalapril as the most prescribed medication; 41% of patients received ARB group medications. There was a higher use of losartan than other medications of this therapeutic group. Ten percent of the patients received angiotensin receptor neprilysin inhibitor (ARNI) at baseline. In total, 85.2% of the patients were receiving some type of RAAS inhibitor (ACEi, ARB, or ARNI).

Mineralocorticoid receptor antagonists (MRA) were prescribed in 59% of the patients at baseline. Spironolactone was the most used medication of this type (1,091 patients). Of the patients who had a reduced ejection fraction (i.e., LVEF ≤40%), 74.6% were taking an MRA.

Of the patients with HFrEF classified as NYHA class I, 82.2% received some type of RAAS, while from the symptomatic (NYHA class II–IV) patients with HFrEF, 86.9% received a RAAS inhibitor, and 88.8% were prescribed a beta-blocker. Only 12.1% of patients with symptomatic HFrEF had been prescribed an ARNI at baseline. From the patients with HFrEF (NYHA class I–IV) 86.3% were receiving some type of RAAS.

Other therapeutic groups were present at baseline; the most relevant were statins and antiplatelet medication, in 52.2% and 46.8% of patients, respectively.

An implantable cardioverter-defibrillator was used by 11.3% of patients, while cardiac resynchronization treatment was used by 8.4% of patients who met criteria for received a device. Table 7 shows the usage of each type of implantable device at baseline.

**Table 7:** Implantable device use at baseline—RECOLFACA registry.


IMPLANTABLE DEVICE	COLOMBIA	ANDEAN	PACIFIC	CARIBBEAN	AMAZON	ORINOCO

**ICD; *N* (%)**	181 (8.9)	134 (11.8)	24 (7.2)	10 (2.5)	12 (7.6)	1 (6.2)

**Dual chamber pacemaker; *N* (%)**	80 (3.9)	54 (4.8)	14 (4.2)	8 (2.0)	4 (2.5)	0 (0)

**Single chamber pacemaker; *N* (%)**	33 (1.6)	20 (1.8)	5 (1.5)	6 (1.5)	2 (1.3)	0 (0)

**CRT-D; *N* (%)**	101 (4.9)	60 (5.3)	24 (7.2)	17 (4.2)	0 (0)	0 (0)

**CRT-P; *N* (%)**	39 (1.9)	24 (2.1)	11 (3.3)	4 (1.0)	0 (0)	0 (0)


ICD: Implantable cardioverter-defibrillator; CRT-D: cardiac resynchronization therapy, defibrillator; CRT-P: cardiac resynchronization therapy, pacemaker.

## Discussion

This study aimed to describe the demographic and clinical characteristics of patients from the RECOLFACA registry. Furthermore, it sought to explore the effect of HF on quality of life and establish healthcare resource utilization patterns in Colombia. The most common etiologies of HF were ischemic and hypertensive heart disease. Hypertension, diabetes, and dyslipidemia were the most prevalent comorbidities. At baseline, around half of the patients had mild symptoms and slight limitations for ordinary activities (NYHA class II), with most patients suffering from HF with a reduced ejection fraction (LVEF ≤40%). At the six-month follow-up, there was an improvement of the LVEF of 2.4%, while 27.4% of patients had a better functional class. The average quality of life score measured using the EQ-5D-3L questionnaire increased by 3.6 points at the six-month follow-up. The mortality rate of patients from the registry was 6.7%.

Regarding their demographic characteristics, patients from the RECOLFACA registry were on average younger than patients from other registry-based studies conducted worldwide [2]. The average age of patients from each region in the country was variable, which may be related to differences in the etiology of heart failure and differences in capabilities for the timely diagnosis of HF. These may be related to the disparity in access to health services between Colombian regions [15]. Additionally, of the 16 patients included for the Orinoco region, 37.5% had missing data at follow-up regarding functional class and QoL, which can explain the difference in mean changes from baseline compared to other regions. As in other registries, most patients were male, which can be explained by the higher lifetime risk of HF in men [16]. Furthermore, most patients had a mixed race or ethnic background and low education levels. Even though socioeconomic status wasn’t directly measured, the type of health insurance is an indirect way of analyzing this variable, and 57.6% of patients belonged to the contributory regime, which suggests they had higher income than their counterparts from the subsidized regime; studies show that they also have better access to health services [7,9].

As in other Latin American countries, the most common etiology of HF was ischemic heart disease [1]. Other causes frequently identified in Latin American populations, such as hypertensive cardiomyopathy, idiopathic dilated cardiomyopathy, and valvular cardiomyopathy, were also frequent in the RECOLFACA registry.

Chagas disease is one of the most mentioned etiologies in studies carried out in LA, accounting for between 8.1 and 21% of the cases of HF in the region [17]. This condition is endemic in Latin American countries, and the Chagas cardiomyopathy associated with this disease has a worse prognosis. Hence, Chagas disease represents a high burden for health systems due to hospital readmissions and mortality [18]. However, in this study, only 3.4% of the patients had been identified as having HF of Chagasic origin. Further research is necessary to understand whether the prevalence of Chagasic disease in Colombia is lower than in other Latin American countries. Other possible causes for this difference may be the underdiagnosis of Chagas or selection bias in the registry, as patients with HF of Chagasic origin may not be included in the registry because they are not treated at the institutions that are part of it.

The current study found that patients from the RECOLFACA registry had a lower hospitalization rate than the estimated hospitalization rates in the Latin American context [1]. The six-month mortality in RECOLFACA was also lower than recently reported for other studies conducted in LA [4]. A possible explanation for these differences might be that the follow-up period of this study was shorter than that of other Latin American studies. The RECOLFACA registry may have patients who are younger and have a more favorable risk profile. Also, better outcomes in this registry compared to other studies might be explained as all patients were actively enrolled in healthcare.

Of the symptomatic patients with HFrEF, a high proportion received a RAAS inhibitor, a beta-blocker, and an MRA. This is an important finding given that, according to European Society of Cardiology guidelines, RAAS inhibitors, beta-blockers, and MRA are the cornerstone of HFrEF treatment [19]. However, at baseline, only a small proportion of the patients who had HFrEF received an ACEi. Most patients classified as NYHA class I with HFrEF had not been prescribed an ACEi previously. The lack of prescription of ACEi for these patients is a relevant finding, as in asymptomatic patients with a reduced LVEF taking ACEi can reduce the risk of requiring hospitalization [19,20,21,22].

Furthermore, 12.4% of the patients with HFrEF had been prescribed an ARNI at baseline, and 24.2% had been prescribed an ARNI at the six-month follow-up. The prescription of ARNI is of particular relevance because in patients with symptomatic HFrEF, the ARNI sacubitril/valsartan reduces the risk of death (cardiovascular and from any cause) or hospitalization for heart failure compared to management with enalapril [23].

Data available for the six-month follow-up showed an increase in the use of all therapeutic groups, but for some therapeutic groups, patients still appeared to be under-prescribed. An under-prescription of HF medications recommended by clinical practice guidelines has been reported in studies conducted in multiple regions of the world [24,25]. Insufficient HF treatment, including not using the appropriate medication and prescribing lower doses than recommended, leads to higher hospitalization rates, decreased quality of life, and higher morbidity and mortality [26]. Interventions that improve adherence to clinical practice guidelines may reduce the burden of HF in Colombia.

The use of high-voltage devices such as defibrillator and resynchronization therapy at baseline were similar to those of large trials at baseline [23]. However, these proportions are low given the proportion of patients with HF who have an indication for these devices [27]. This is an important finding considering that these devices are financed by the Colombian health system [28].

The findings of this study may be somewhat limited by a large number of missing data. Because the aim was to describe the current clinical practice, diagnostic tests were not conducted in a standardized manner at each data collection point. LVEF was available for only 763 patients at the six-month follow-up. Moreover, as patients with missing data for relevant clinical characteristics at baseline such as LVEF, stage of heart failure, and NYHA Functional Classification were excluded from the analyses, only 2,045 of the 2,528 patients from the registry were included in this study. It is vital to bear in mind that the exclusion of these patients may have caused selection bias, given that patients for whom fewer data were available might be receiving inferior care and have consequently poorer outcomes.

Further efforts are necessary to broaden the reach of the RECOLFACA registry in the country. Moreover, a more consistent collection of patient information may strengthen the registry’s capability to provide valuable insights regarding HF in the country.

Notwithstanding these limitations, the current study fills critical evidence gaps regarding the demographic and clinical characteristics, QoL, and healthcare resource utilization patterns of patients with HF in Colombia.

## Conclusion

The use of information from the RECOLFACA registry for this study allowed for a characterization of patients with heart failure in Colombia, where we found the most common etiology was ischemic heart disease, with hypertension, diabetes, and dyslipidemia as the most relevant comorbidities. Although the results of this study show that multiple evidence-based treatments for heart failure are being widely used in Colombia, there seems to be room for improvement regarding some interventions for the treatment of patients with heart failure. Furthermore, the registry allowed the possibility to analyze HCRU by Colombian regions, which can allow further studies to allocate resources based on the distribution of etiologies, medication use, and hospitalization requirements. More studies must be conducted to identify risk factors for HF for our population, and registries are a step forward in helping solve this sort of knowledge gaps.

## Additional File

The additional file for this article can be found as follows:

10.5334/gh.1145.s1 Supplementary Materials. Supplementary Table 1.

## References

[1] Ciapponi A, Alcaraz A, Calderón M, Matta MG, Chaparro M, Soto N, et al. Carga de enfermedad de la insuficiencia cardiaca en América Latina: Revisión sistemática y metanálisis. Revista Espanola de Cardiologia. 2016; 69(11): 1051–1060. DOI: 10.1016/j.recesp.2016.04.045 27553287

[2] Ambrosy AP, Fonarow GC, Butler J, Chioncel O, Greene SJ, Vaduganathan M, et al. The global health and economic burden of hospitalizations for heart failure: Lessons learned from hospitalized heart failure registries. JACA. 2014; 63(12): 1123–1133. DOI: 10.1016/j.jacc.2013.11.053 24491689

[3] Taylor CJ, Ryan R, Nichols L, Gale N, Hobbs FR, Marshall T. Survival following a diagnosis of heart failure in primary care. Family practice. 2017; 34(2): 161–168. DOI: 10.1093/fampra/cmw145 28137979 PMC6192063

[4] Tromp J, Bamadhaj S, Cleland JGF, Angermann CE, Dahlstrom U, Ouwerkerk W, et al. Correction to: Post-discharge prognosis of patients admitted to hospital for heart failure by world region, and national level of income and income disparity (REPORT-HF): A cohort study. Lancet Glob Health. 2020; 8(8): e1001. DOI: 10.1016/S2214-109X(20)30294-1 32710857

[5] Olivera MJ, Fory JA, Porras JF, Buitrago G. Prevalence of Chagas disease in Colombia: A systematic review and meta-analysis. PloS one. 2019; 14(1): e0210156-e. DOI: 10.1371/journal.pone.0210156 30615644 PMC6322748

[6] Instituto Nacional de Salud, Observatorio Nacional de Salud. Acceso a servicios de salud en Colombia: Décimo primer Informe Técnico. Acceso a servicios de salud en Colombia; 2019. (accessed 3 June 2022).

[7] Montenegro Torres F, Bernal Acevedo O. Colombia case study: The subsidized regime of Colombia’s national health insurance system. UNICO Studies Series. 2013; p. 15. https://openknowledge.worldbank.org/handle/10986/13285 (accessed 1 June 2022).

[8] Colombia MdSyPS. Cifras de aseguramiento en salud. Mibisterio de Salud; 2021. https://www.minsalud.gov.co/proteccionsocial/Paginas/cifras-aseguramiento-salud.aspx.

[9] Garcíai JA. La salud en Colombia: Más cobertura pero menos acceso. Documentos de Trabajo Sobre Economía Regional. 2014; p. 204. (accessed 3 June 2022).

[10] Colombia MdSyPS. Servicios y tecnologías de salud financiados con recursos de la Unidad por Pago por Capitación (UPC). Resolución Número 2292. Ministerio de Salud; 2021. https://www.minsalud.gov.co/Normatividad_Nuevo/Resoluci%C3%B3n%20No.%202292%20de%202021.pdf.

[11] Diaztagle-Fernández JJ, Latorre-Alfonso SI, Maldonado-Arenas SE, Manosalva-Álvarez GP, Merchán-Cepeda JS, Centeno-García CD, et al. Research on heart failure in Colombia 1980–2015: A systematic review. Revista Facultad de Medicina. 2018; 66(2): 139–351. DOI: 10.15446/revfacmed.v66n2.60005

[12] Mora-Pabón G. Research on heart failure in Colombia, time to take a step forward. Revista Facultad de Medicina. 2018; 66(2): 137–138. DOI: 10.15446/revfacmed.v66n2.70828

[13] EuroQol Research Foundation. EQ-5D-3L user guide. https://euroqol.org/publications/user-guides2018.

[14] The R Foundation. The R project for statistical computing. Vienna; 2020.

[15] Garcia-Ramirez J, Nikoloski Z, Mossialos E. Inequality in healthcare use among older people in Colombia. International Journal for Equity in Health. 2020; 19(1): 168. DOI: 10.1186/s12939-020-01241-0 33100214 PMC7646194

[16] Pandey A, Omar W, Ayers C, LaMonte M, Klein L, Allen NB, et al. Sex and race differences in lifetime risk of heart failure with preserved ejection fraction and heart failure with reduced ejection fraction. Circulation. 2018; 137(17): 1814–1823. DOI: 10.1161/CIRCULATIONAHA.117.031622 29352072 PMC6417883

[17] Bocchi EA, Arias A, Verdejo H, Diez M. The reality of heart failure in Latin America. 2013; 62(11). DOI: 10.1016/j.jacc.2013.06.013 23850910

[18] Echeverría LE, Marcus R, Novick G, Sosa-Estani S, Ralston K, Zaidel EJ, et al. WHF IASC roadmap on Chagas disease. Glob Heart. 2020; 15(1): 26. DOI: 10.5334/gh.484 32489799 PMC7218776

[19] Ponikowski P, Voors AA, Anker SD, Bueno H, Cleland JG, Coats AJ, et al. 2016 ESC guidelines for the diagnosis and treatment of acute and chronic heart failure: The task force for the diagnosis and treatment of acute and chronic heart failure of the European Society of Cardiology (ESC). Developed with the special contribution of the Heart Failure Association (HFA) of the ESC. Eur J Heart Fail. 2016; 18(8): 891–975. DOI: 10.1002/ejhf.592 27207191

[20] Pfeffer MA, Braunwald E, Moyé LA, Basta L, Brown EJ, Jr., Cuddy TE, et al. Effect of captopril on mortality and morbidity in patients with left ventricular dysfunction after myocardial infarction. Results of the survival and ventricular enlargement trial. The SAVE Investigators. N Engl J Med. 1992; 327(10): 669–677. DOI: 10.1056/NEJM199209033271001 1386652

[21] The SOLVD Investigators. Effect of enalapril on mortality and the development of heart failure in asymptomatic patients with reduced left ventricular ejection fractions. New England Journal of Medicine. 1992; 327(10): 685–691. DOI: 10.1056/NEJM199209033271003 1463530

[22] Jong P, Yusuf S, Rousseau MF, Ahn SA, Bangdiwala SI. Effect of enalapril on 12-year survival and life expectancy in patients with left ventricular systolic dysfunction: A follow-up study. Lancet. 2003; 361(9372): 1843–1848. DOI: 10.1016/S0140-6736(03)13501-5 12788569

[23] McMurray JJV, Packer M, Desai AS, Gong J, Lefkowitz MP, Rizkala AR, et al. Angiotensin-neprilysin inhibition versus enalapril in heart failure. New England Journal of Medicine. 2014; 371(11): 993–1004. DOI: 10.1056/NEJMoa1409077 25176015

[24] Maggioni AP, Dahlström U, Filippatos G, Chioncel O, Leiro MC, Drozdz J, et al. EURObservational Research Programme: Regional differences and 1-year follow-up results of the Heart Failure Pilot Survey (ESC-HF Pilot). European Journal of Heart Failure. 2013; 15(7): 808–817. DOI: 10.1093/eurjhf/hft050 23537547

[25] Gastelurrutia P, Benrimoj SI, Espejo J, Tuneu L, Mangues MA, Bayes-Genis A. Negative clinical outcomes associated with drug-related problems in heart failure (HF) outpatients: Impact of a pharmacist in a multidisciplinary HF clinic. Journal of Cardiac Failure. 2011; 17(3): 217–223. DOI: 10.1016/j.cardfail.2010.10.009 21362530

[26] Wiggins BS, Rodgers JE, DiDomenico RJ, Cook AM, Page II RL. Discharge counseling for patients with heart failure or myocardial infarction: A best practices model developed by members of the American College of Clinical Pharmacy’s Cardiology Practice and Research Network based on the Hospital to Home (H2H) Initiative. Pharmacotherapy. 2013; 33(5): 558–580. DOI: 10.1002/phar.1231 23529897

[27] van Veldhuisen DJ, Maass AH, Priori SG, Stolt P, van Gelder IC, Dickstein K, et al. Implementation of device therapy (cardiac resynchronization therapy and implantable cardioverter defibrillator) for patients with heart failure in Europe: Changes from 2004 to 2008. Eur J Heart Fail. 2009; 11(12): 1143–1151. DOI: 10.1093/eurjhf/hfp149 19884129

[28] Ministerio de Salud y Protección Social, Colombia. Listado Actualización Integral 2020–2021; 2021.

