# Supplementary material for: Characteristics and Outcomes of Heart Failure Patients from a Middle-Income Country: The RECOLFACA Registry
Source: Glob Heart. 2022 Aug 18;17(1):57. doi: 10.5334/gh.1145 (PMC9389953; doi:10.5334/gh.1145)
Supplement: Supplementary Materials. — Supplementary Table 1. [file gh-17-1-1145-s1.pdf]

Supplementary Materials

Supplementary Table 1:

	Baseline (*N* = 2,045)						6-month follow-up (*N* = 1,907*)					
	**Colombia**	**Andean**	**Pacific**	**Orinoco**	**Caribbean**	**Amazon**	**Colombia**	**Andean**	**Pacific**	**Orinoco**	**Caribbean**	**Amazon**
**Hb (mg/dL)**												
**Mean ± SD**	12.9 ± 2.1	13.2 ± 2.1	12.8 ± 2.0	14.0 ± 1.6	12.2 ± 1.8	12.5 ± 2.2	13.1 ± 1.9	13.4 ± 1.8	13.0 ± 1.9	13.4 ± 0.9	12.3 ± 1.7	12.8 ± 2.3
**Missing data; *N* (%)**	472 (23.1)	212 (18.7)	109 (32.5)	5 (31.3)	118 (29.1)	28 (17.8)	955 (50.1)	471 (45)	209 (65.9)	12 (75)	213 (55.3)	50 (35)
**Blood creatinine (mg/dL)**												
**Mean ± SD**	1.3 ± 1.0	1.3 ± 1.0	1.2 ± 0.6	1.0 ± 0.3	1.4 ± 1.2	1.6 ± 1.4	1.5 ± 3.4	1.5 ± 4.0	1.5 ± 2.3	1.3 ± 0.5	1.6 ± 2.1	1.7 ± 1.3
**Missing data; *N* (%)**	334 (16.3)	140 (12.4)	66 (19.7)	4 (25)	91 (22.5)	33 (21)	868 (45.5)	403 (38.5)	184 (58))	11 (68.8)	205 (53.3)	65 (45.5)
**GFR (mL/min)**												
**Mean ± SD**	63.3 ± 34.8	62.6 ±29.7	66.0 ±48.9	78.4 ± 30.7	65.3 ±37.5	56.5 ±27.7	61.1 ±33.3	61.9 ±34.9	61.2 ± 28.1	55.9 ± 21.7	62.3 ±34.3	51.9 ±25.1
**Missing data; *N* (%)**	334 (16.3)	140 (12.4)	66 (19.7)	4 (25)	91 (22.5)	33 (21)	868 (45.5)	403 (38.5)	184 (58))	11 (68.8)	205 (53.3)	65 (45.5)
**K+ mEq/L *N* (%)**												
**<3.5**	71 (3.5)	36 (3.2)	6 (1.8)	0 (0.0)	19 (4.7)	10 (6.4)	55 (2.9)	41 (3.9)	1 (0.3)	0 (0)	9 (2.3)	4 (2.8)
**3.5–5**	1,354 (66.2)	785 (69.3)	229 (68.4)	10 (62.5)	247 (61.0)	83 (52.9)	806 (42.3)	494 (47.2)	114 (36)	5 (31.3)	152 (39.5)	41 (28.7)
**>5**	143 (7.0)	87 (7.7)	12 (3.6)	1 (6.2)	33 (8.1)	10 (6.4)	102 (5.3)	63 (6)	14 (4.4)	0 (0)	14 (3.6)	11 (7.7)
**Unknown**	477 (23.3)	224 (19.8)	88 (26.3)	5 (31.2)	106 (26.2)	54 (34.4)	944 (49.5)	448 (42.83)	188 (59.31)	11 (68.75)	210 (54.55)	87 (60.84)
**Na+ mEq/L *N* (%)**												
**<135**	192 (9.4)	95 (8.4)	14 (4.2)	1 (6.2)	46 (11.4)	36 (22.9)	114 (6.0)	59 (5.6)	7 (4.9)	0 (0)	41 (10.6)	7 (2.2)
**>135**	1,245 (60.9)	744 (65.7)	213 (63.6)	10 (62.5)	216 (53.3)	62 (39.5)	731 (38.3)	470 (44.9)	101 (31.9)	5 (31.3)	111 (28.8)	44 (30.8)
**Missing data; *N* (%)**	608 (29.7)	293 (25.9)	108 (32.2)	5 (31.2)	143 (35.3)	59 (37.6)	1,062 (55.7)	517 (49.4)	209 (65.9)	11 (68.8)	233 (60.5))	71 (4.4)

**BUN (mg/dL)**												
**Mean ± SD**	26.4 ± 13.8	27.7 ± 14.1	25.5 ± 12.2	22.5 ± 10.8	22.9 ± 13.0	26.3 ± 15.1	25.0 ± 14.0	25.3 ± 13.5	25.3 ± 15.0	31.6 ± 16.0	23.3 ± 13.7	26.8 ± 16.6
**Missing data; *N* (%)**	760 (37.2)	361 (31.9)	168 (50.1)	5 (31.3)	176 (43.5)	50 (31.8)	279 (14.6)	118 (11.3)	62 (19.6)	6 (37.5)	72 (18.7)	21 (14.7)
**Glycemia mg/dl; *N* (%)**												
**<100**	557 (27.2)	316 (27.9)	89 (26.6)	2 (12.5)	126 (31.1)	24 (15.3)	322 (16.9)	188 (18)	40 (12.6)	1 (6.3)	82 (21.3)	11 (7.7)
**100–125**	404 (19.8)	206 (18.2)	60 (17.9)	3 (18.8)	89 (22.0)	46 (29.3)	273 (14.3)	174 (16.6)	30 (9.5)	2 (12.5)	47 (12.2)	20 (14)
**>125**	221 (10.8)	102 (9.0)	20 (6.0)	2 (12.5)	61 (15.1)	36 (22.9)	109 (5.7)	53 (5.1)	9 (2.8)	1 (6.3)	24 (6.2)	22 (15.4)
**Missing data; *N* (%)**	863 (42.2)	508 (44.9)	166 (49.6)	9 (56.2)	129 (31.9)	51 (32.5)	1,203 (63.1)	631 (60.3)	238 (75.1)	12 (75)	232 (60.3)	90 (62.9)
